# Association of periodontitis and complications after pancreatic surgery

**DOI:** 10.1093/bjsopen/zrad050

**Published:** 2023-10-05

**Authors:** Kethe M Hermunen, Tiina Vuorela, Johanna M Louhimo, Inkeri Lehto, Jaana Hagström, Caj H Haglund, Timo Sorsa, Hanna E Seppänen

**Affiliations:** Department of Surgery, University of Helsinki and Helsinki University Hospital, Helsinki, Finland; Department of Surgery, University of Helsinki and Helsinki University Hospital, Helsinki, Finland; Department of Surgery, University of Helsinki and Helsinki University Hospital, Helsinki, Finland; Department of Radiology, HUS Diagnostic Center, University of Helsinki and Helsinki University Hospital, Helsinki, Finland; Department of Pathology, University of Helsinki and Helsinki University Hospital, Helsinki, Finland; Department of Oral Pathology and Radiology, University of Turku, Turku, Finland; Translational Cancer Medicine Research Program, Faculty of Medicine, University of Helsinki, Helsinki, Finland; Department of Surgery, University of Helsinki and Helsinki University Hospital, Helsinki, Finland; Translational Cancer Medicine Research Program, Faculty of Medicine, University of Helsinki, Helsinki, Finland; iCAN Digital Precision Cancer Medicine Flagship, Helsinki, Finland; Department of Oral and Maxillofacial Diseases, Helsinki University Hospital, Helsinki, Finland; Department of Periodontology, Karolinska Institutet, Huddinge, Sweden; Department of Surgery, University of Helsinki and Helsinki University Hospital, Helsinki, Finland; Translational Cancer Medicine Research Program, Faculty of Medicine, University of Helsinki, Helsinki, Finland; iCAN Digital Precision Cancer Medicine Flagship, Helsinki, Finland

## Introduction

Periodontitis, a chronic infection of the tooth-supporting structures, has a worldwide prevalence of 45 to 50 per cent, with the severe form affecting 10 to 15 per cent^1^. In Finland, the prevalence is 20 to 27 per cent^2^.

Severe periodontitis associates with diabetes^3^, cardiovascular disease^4^, chronic obstructive pulmonary disease^5^, chronic kidney disease^6^, and chronic liver disease^7^. Periodontitis bacteria can enter the blood circulation and trigger inflammatory conditions and thus promote malignancies^8^.

Pancreatic surgery carries a high risk of potentially life-threatening complications: postoperative pancreatic fistula (POPF), delayed gastric emptying (DGE), postpancreatectomy haemorrhage (PPH), and infections^9^.

Matrix metalloproteinases (MMPs) increase in tissues due to pathological conditions resulting from inflammation, infection, and malignant transformation^10^. Elevated MMP-8 reflects the clinical diagnosis, course, and severity of periodontitis^10^.

Periodontitis is traditionally diagnosed by clinical examinations. Recent studies show that periodontitis can conveniently be diagnosed from mouthrinse using an active MMP-8 point-of-care test (aMMP-8 POCT)^11^. Periodontitis has previously been associated with a risk of postoperative complications after cardiac surgery^12,13^.

The aim of this study was to investigate the prevalence of periodontitis in patients undergoing pancreatic surgery, and whether periodontitis elevates the risk of postoperative complications.

## Methods

This was a prospective study conducted at Helsinki University Hospital between May 2017 and June 2019. Before surgery, patients eligible for pancreatic surgery were screened for periodontitis.

Information collected comprised age, sex, BMI, smoking, diabetes, tumour histology, type of surgery, the ASA physical status classification, and postoperative complications for a 30-day interval. Pancreatectomy-specific complications were classified according to the consensus statement of the International Study Group for Pancreatic Fistula (ISGPF) and the International Study Group in Pancreatic Surgery (ISGPS), and general complications in accordance with the Clavien–Dindo classification.

The main pancreatic duct diameter was measured at the level of the portal confluence at preoperative images (MRI or CT) by a dedicated radiologist, classifying it as greater than 3 or less than or equal to 3 mm.

The mouthrinse aMMP-8 POCT assesses elevated (greater than 20 ng/ml) levels of active—not total—MMP-8 in oral fluids. The sensitivity and specificity of the aMMP-8 POCT are 75 to 85 and 80 to 90 per cent^11^. The aMMP-8 POCT technology has been validated worldwide to diagnose and screen for periodontitis^11^. A trained nurse performed the test according to the manufacturer’s instructions. The test-kit manufacturers (Medix Biochemical Ltd, Espoo, Finland, and Dentognostics GmbH, Jena, Germany) took no part in the study and did not have any financial involvement.

Statistical analysis was performed using SPSS^®^ (IBM, Armonk, NY, USA; version 26), and the associations of categorical variables were assessed using a chi-squared test and Fisher’s exact test when applicable. Logistic regression allowed assessment on complications in a multivariate setting. *P* <0.050 was considered statistically significant.

The Ethics Committee of Helsinki University Hospital approved the study (HUS 435/2017). Informed consent was a requirement of all patients. This prospective study is registered at http://www.clinicaltrials.gov (NCT03144973).

## Results

A total of 220 consecutive patients were screened for eligibility and 26, whose operation was cancelled, were excluded (*Table 1*). Some 68 (35 per cent) patients were affected by periodontitis.

**Table 1:** Patient characteristics and their associations with periodontitis

	All, *n* = 194	Periodontitis	No periodontitis	*P*
**Sex**				0.259
Female	92 (47)	36 (39)	56 (61)	
Male	102 (53)	32 (31)	70 (69)	
**Age (years)**				0.140
<55	36 (19)	8 (22)	19 (78)	
55–70	89 (46)	32 (36)	57 (64)	
71–85	69 (36)	28 (41)	41 (59)	
**Histology**				0.155
Benign	52 (27)	13 (25)	39 (75)	
Malignant	142 (73)	55 (39)	87 (61)	
**Procedure**				0.240
Whipple	125 (64)	43 (34)	82 (66)	
Distal pancreatectomy	55 (28)	21 (38)	35 (62)	
Total pancreatectomy	6 (3)	3 (50)	3 (50)	
Other*	8 (4)	1 (14)	6 (86)	
PDVR	46 (24)	16 (35)	30 (65)	
**Patient-related factors**				
Obesity (BMI >30 kg/m^2^)	31 (16)	15 (48)	16 (52)	0.081
Diabetes (missing values = 16)	29 (15)	12 (41)	17 (59)	0.419
Smoking	28 (14)	12 (43)	16 (57)	0.351
**Pancreas-related factors**				
Pancreatic duct diameter				0.122
≤3 mm	114 (59)	35 (31)	79 (69)	
>3 mm	76 (39)	32 (42)	44 (58)	
Missing values	4 (2)			
Gland texture				0.860
Soft	77 (40)	28 (36)	49 (64)	
Firm	62 (32)	24 (39)	38 (61)	
Missing values	55 (28)			

Values are *n* (%). *Modified pancreas resection, pancreas enucleation, or Appleby procedure. PDVR, pancreatoduodenectomy with vascular reconstruction.

Postoperative thrombotic events and PPH associated significantly with periodontitis (*P* = 0.018 and *P* = 0.004 respectively) (*Table 2*). Patients with periodontitis had grade B–grade C PPH significantly more frequently, which did not associate with clinically relevant POPF. Assessed using logistic regression, and adjusting for age, sex, smoking, diabetes, obesity, and ASA grade, the sole independent risk factor for thrombotic events was periodontitis (*P* = 0.022), with OR 2.83 (95 per cent c.i. 1.17 to 6.88). For PPH, periodontitis was an independent risk factor (*P* = 0.003), with OR 3.63 (95 per cent c.i. 1.54 to 8.55), as was male sex (*P* = 0.026), with OR 2.80 (95 per cent c.i. 1.13 to 6.97).

**Table 2:** Postoperative complications in patients with and without periodontitis

	All	Periodontitis	No periodontitis	*P*
**Postoperative thrombotic events**				
All patients	24 (12)	14 (58)	10 (42)	0.018
Whipple	16	10	6	0.016
Distal pancreatectomy	7	3	4	1.000
Deep-vein thrombosis	6 (3)	3 (50)	3 (50)	0.425
Pulmonary embolism	14 (7)	8 (57)	6 (43)	0.086
**PPH**				
All patients				0.004
None–grade A	167 (86)	44 (35)	105 (65)	
Grade B–grade C	27 (14)	24 (53)	21 (47)	
Whipple				0.310
None–grade A	105 (84)	34 (32)	71 (68)	
Grade B–grade C	20 (16)	9 (45)	11 (55)	
Distal pancreatectomy				0.018
None–grade A	51 (93)	17 (33)	34 (67)	
Grade B–grade C	4 (7)	4 (100)	0 (0)	
POPF grade B–grade C	17 (9)	4 (24)	13 (76)	0.426
DGE grade B–grade C	29 (15)	7 (24)	22 (76)	0.211
Chyle leak	13 (7)	6 (46)	7 (54)	0.384
Postoperative infections, all*	61 (31)	23 (38)	38 (62)	0.628
Urinary	6 (3)	2 (33)	4 (67)	1.000
Pneumonia	25 (14)	6 (24)	19 (76)	0.265
Clavien–Dindo grade 0–grade 2	139 (71)	46 (33)	93 (54)	0.401
Clavien–Dindo grade 3–grade 5	54 (28)	22 (54)	32 (46)	

Values are *n* (%). Associations were assessed using a chi-squared test or Fisher’s exact test. *Including nine wound infections and 39 intra-abdominal infections. PPH, postpancreatectomy haemorrhage; POPF, postoperative pancreatic fistula; DGE, delayed gastric emptying.

In a sub-analysis of patients undergoing Whipple procedures, only postoperative thrombotic events associated with periodontitis (*P* = 0.016). After adjustment for patient-related factors, the only significant independent risk factor was periodontitis (*P* = 0.021), with OR 3.75 (95 per cent c.i. 1.22 to 11.50).

In a sub-analysis of distal pancreatectomies, only PPH associated with periodontitis (*P* = 0.018), and the patients without periodontitis experienced significantly less postoperative haemorrhage. All grade B–grade C PPH cases were in the periodontitis group. In the multivariate logistic regression model for PPH, the number of patients was too small for any of the variables to reach significance.

The frequencies of other postoperative complications (POPF, DGE, chyle leak, and postoperative infections) and all surgical and non-surgical complications showed no differences between patients with or without periodontitis (*Table 2*).

## Discussion

This study is, to the best of the authors' knowledge, the first to evaluate the prevalence of periodontitis among patients undergoing gastrointestinal surgery and to evaluate the impact of periodontitis on postoperative complications.

Periodontitis prevalence in patients undergoing pancreatic surgery was 35 per cent. Postoperative thrombotic events and PPH were more common in patients with periodontitis, whereas no differences emerged in POPF, in DGE, in chyle leak, or in postoperative infections. For thrombotic events, the sole independent risk factor was periodontitis.

Inflammation is a common pathway to the triggering of venous thromboembolism (VTE)^14^. Periodontitis may promote VTE risk through infection-induced systemic inflammation contributing to platelet aggregation and hypercoagulability^15^. The relationship between periodontitis and VTE is, however, not very well understood. Sanchez-Siles *et al*.^16^ found periodontitis prevalence to be higher in patients with VTE than in those without (73 *versus* 45 per cent respectively), and found a VTE risk nearly 2-fold in patients with periodontitis than in those without. Self-reported tooth loss due to periodontitis associated with a 30 per cent higher VTE risk, but there was no association between periodontitis and VTE^15^.

Patients with periodontitis suffered significantly more postoperative thrombotic events, similar to the findings of Sanchez-Siles *et al.*^16^, but unlike those of Cowan *et al.*^15^. Notably, neither of these groups studied thrombotic events in a postoperative setting.

Among all patients, PPH associated significantly with periodontitis. With regard to PPH, periodontitis and male sex were independent risk factors.

A literature review revealed no studies on periodontitis and postoperative haemorrhage in abdominal surgery, but two studies revealed an association between impaired oral health and postoperative infections in cardiac surgery^12,13^. This study found no association between periodontitis and postoperative infections in pancreas surgery.

A 10-year Finnish follow-up study showed a clear association between periodontitis and pancreatic cancer mortality^8^. A large Taiwanese database study revealed periodontitis to be a risk factor for pancreatic cancer independent of diabetes, pancreatitis, and alcohol-related conditions^17^. A Swedish nationwide registry-based cohort study confirmed an association between poor oral health and pancreatic cancer risk^18^. Fan *et al*.^19^ showed that carriers of the periodontal pathogens *Porphyromonas gingivalis* or *Aggregatibacter actinomycetemcomitans* had an elevated risk of pancreatic cancer, pointing to a potential aetiological role. This is further supported by immunohistochemical findings revealing oral bacteria-specific enzymes in human pancreatic cancer tissues^20^.

The prospective nature of the study and the fact that surgeons were blinded to the aMMP-8 POCT results are among its strengths. The total number of study patients is adequate, but the somewhat heterogeneous cohort and rather small subgroups are limitations.

Further studies are essential to confirm our finding regarding periodontitis as a risk factor for postoperative thromboembolism and haemorrhage. In addition, further investigation is vital to clarify whether preoperative intervention against periodontitis can reduce postoperative complications.

## Data Availability

Our research data are available upon request.
